# Enhanced Optimization of Composite Laminates: Multi-Objective Genetic Algorithms with Improved Ply-Stacking Sequences

**DOI:** 10.3390/ma17040887

**Published:** 2024-02-15

**Authors:** Ramesh Kumpati, Wojciech Skarka, Michał Skarka, Miha Brojan

**Affiliations:** 1 Department of Fundamentals of Machinery Design, Silesian University of Technology, 44-100 Gliwice, Poland; 2 Faculty of Aerospace Engineering, Delft University of Technology, Kluyverweg 1, 2629 HS Delft, The Netherlands; michal.skarka@gmail.com; 3 Faculty of Mechanical Engineering, University of Ljubljana, SI-1000 Ljubljana, Slovenia; miha.brojan@fs.uni-lj.si

**Keywords:** stacking sequence, composite laminate, optimization, failure criteria, buckling

## Abstract

This study introduces multi-objective genetic algorithms for optimizing the stacking sequence of lightweight composite structures. Notably, significant emphasis is placed on adhering to engineering design guidelines specific to stacking sequence design. These guidelines are effectively integrated into the optimization problem formulation as either constraints or additional objectives. To enhance the initialization process, a novel strategy is proposed based on mechanical considerations. The method is then applied to optimize a composite laminate in terms of weight, inverse reserve factor, and buckling load factor. Three laminates were considered, and the influence of the design and the material composition on their mechanical properties were studied. This research demonstrated that a new stacking sequence [90_6_/45_4_/0_6_] resulted in improved optimum designs compared to the traditional stacking sequence comprising plies at 0°, 45°, and 90° angles. These outcomes can be deemed the optimum stacking sequence, making them valuable for future applications in unmanned aerial vehicle (UAV) structures.

## 1. Introduction

Composite materials are advanced materials that are mainly used in the aerospace industry. The substances can be customized to meet specific needs, such as high strength and lightness [1]. The construction of composite materials was an important advance in materials technologies. Although the initial composite material was invented at the beginning of the 20th century, composites were not widely adopted in industry until the 1960s and 1970s [2]. Since then, there have been many developments in the manufacture and utilization of composite materials. While there are numerous materials on the market today, composites have unique properties based on their intended use. As a result, the evolution and optimization of composite materials are critical.

Synthetic materials used for composites include glass-fiber-reinforced polymer (GFRP), carbon fiber-reinforced polymer (CFRP), and ceramic matrix composites (CMCs) [3]. On the other hand, there are other popular fibers that are derived from naturally occurring sources. These substances have been used for thousands of years for a variety of purposes, including transportation, shelter, and clothing. Natural fibers have gained popularity in a variety of industries over the last decade, including the automotive, textile, and aerospace construction industries, due to their distinct properties and environmental friendliness [4,5]. Mineral fibers, plant fibers, and animal fibers are the three types of natural fibers. Linen, cotton, and jute are plant fibers popular in the textile industry due to their breathability, durability, and softness. Silk and wool, for example, are well known for their durability and are commonly recognized for their softness, warmth, and natural moisture-wicking properties. Mineral fibers, including rock wool and asbestos, are employed for fire resistance and insulation [6,7,8]. Our current research focuses on the optimization of composite laminate parts, regardless of the type of fibers used. Ply-stacking sequence design in composite laminates is typically a combinatorial problem, with limited ply thicknesses and ply orientations available at specific ply angles. These local design challenges are closely interconnected with the overall structure’s design. The global optimization rank imposes constraints on individual panel designs, often specifying the geometrical dimensions of the panel. The selected material, a set of design loads, and an initial evaluation determine the required number of angles, such as 0°, ±45°, and 90° plies.

Numerous studies have been conducted and published on composite structure optimization during the last few decades. Venkatraman et al. [9] conducted a detailed review of the optimization of composite laminates and stiff-end panels. From these studies, genetic algorithms (GAs) stand out as one of the most important and popular methods for investigating optimization problems. These algorithms are well suited for permutation problems and provide optimum designs for designers. Although the stacking sequence arrangement may only have slight variations, it can significantly impact the overall performance of the laminate configuration. Most studies have focused solely on single-objective optimization methodologies. For example, some aimed to minimize the total number of plies in the composite laminate [10], while others aimed to maximize the buckling load [11]. Only a few studies have stared at the current optimization aspect, specifically the multi-objective approach. These have tried to find the best balance between the stiffened panel’s mass and total cost [12] or between its mass and how much it bends under a bending load [13]. Other studies have also addressed the optimization method, such as investigating Pareto-based GAs [14,15,16,17,18,19,20]. Industries in the aeronautics field are currently facing a significant workload in the optimization process due to numerous load cases. The number of load cases can exceed hundreds, resulting in an increase in objective functions and corresponding constraints. Still, the optimization of composite structures is rarely considered a solution to this problem [21].

**Figure 1:**
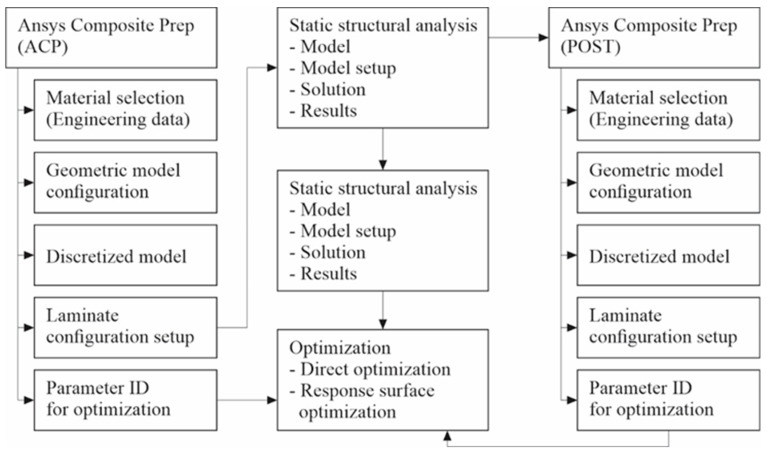
Multi objective optimization layout.

The optimization of the composite structure consists of five main stages. The input module, such as, e.g., Ansys Composite Prep (ACP), usually consists of five parts: material selection, geometric model, discretized model, laminate configuration, and parameter selection. The laminate configuration part is connected to the second module, which is static structural analysis. This module is further subdivided into four parts: model, model setup, solution, and results. This static analysis is linked to the third stage, which is eigen buckling analysis, utilizing, e.g., Ansys Composite Pre (POST) and the optimization results include both direct optimization and response surface optimization. A general description of the multi-objective evolutionary strategy which is the focus of this study is provided as a block diagram in Figure 1. In our case, the analysis is then performed using the ANSYS Workbench, while also addressing the direct optimization method using genetic algorithms.

Finally, the optimum design of a composite laminate under different load cases can be obtained. The composite laminate is optimized for weight, the inverse reserve factor, and the buckling margin for each load case, following design rules and considering material strength.

## 2. Material Methods

**Figure 2:**
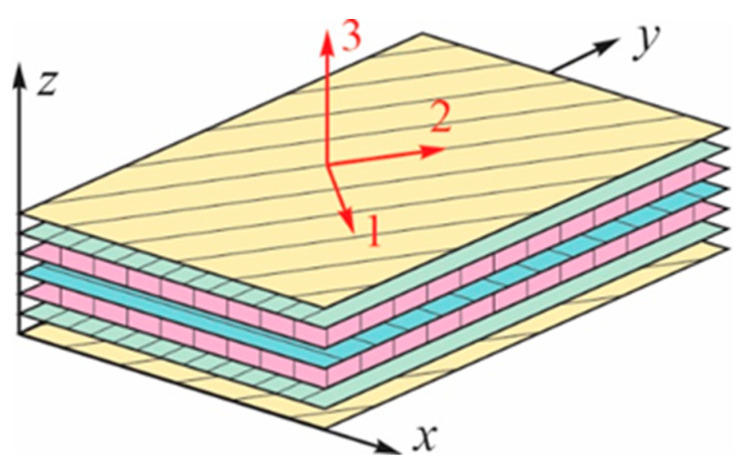
Material coordinate system (1, 2, 3) and global coordinates (*x*, *y*, *z*) on a laminated composite plate.

In the following description, a rectangular Cartesian coordinate system (x, y, z) is used to distinguish the stresses and strains of an N-layer composite laminated plate. A layer-wise material coordinate system (1, 2, 3) is employed to analyze the laminate failure. Axis 1 refers to the fiber direction, and axis 2 refers to the transverse in-plane direction, as shown in Figure 2.

### 2.1. Mechanical Analysis of the General Problem

Following the ideas of classical lamination theory [22,23,24,25], the composite laminate analysis is performed assuming a symmetric stiffness matrix relating forces per unit width (*N*) = (*N_xx_*, *N_yy_*, *N_xy_*) and the moment resultants per unit width of the laminate (*M*) = (*M_xx_*, *M_yy_*, *M_xy_)*, as well as the mid-surface strains (*ɛ*) = (*ɛ_xx_*°, *ɛ_yy_*°, *γ_xy_*°) and curvatures (*k*) *=* (*k_xx_*°, *k_yy_*°, *k_xy_*°). As such,

(1) NM=ABBDɛ°k°

**Figure 3:**
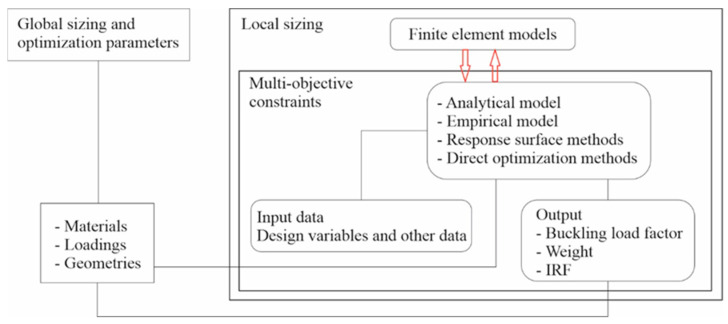
Numerical calculation strategy.

The coefficients of a submatrix *A*, *Aij*, show how stiff the matrix is when stretched (where *i* and *j* range from 1 to 2 to 6 in the engineering notation, where 1 is the direction of the reinforcement (fiber), 2 is the transverse in-plane direction, and 6 is the in-plane (shear). These values change depending on the orientation of the ply and the total thickness. The coefficients *D_ij_* are the matrix bending stiffnesses, which depend on the ply orientation, thickness of the ply, and stacking sequence. The coefficients *B_ij_* are the bending extensional coupling stiffnesses. They are calculated as follows:

(2) $$\begin{array}{c} A=\sum_{k=1}^{N}(z_{k}-z_{k-1\;}{)Q}^{\left(k\right)}\\ B=\frac{1}{2}\sum_{k=1}^{N}(z_{k}^{2}-z_{k-1}^{2}{)Q}^{\left(k\right)}\\ D=\frac{1}{3}\sum_{k=1}^{N}(z_{k}^{3}-z_{k-1}^{3}{)Q}^{\left(k\right)} \end{array}$$

where *z_k_* and *z_k−_*_1_ are the vertical positions of the upper- and lower-direction surfaces of the *k-*th ply orientated with angle *θ_k_*. The overall numerical calculation strategy is shown in Figure 3.

In the component material coordinate system attached to the *k*-th ply, stresses and strains are related as

(3) $$\left(\begin{array}{c} \delta_{1}\\ \delta_{2}\\ \tau_{12} \end{array}\right)=\left(\begin{array}{ccc} {Ǭ}_{11} & {Ǭ}_{12} & 0\\ {Ǭ}_{12} & {Ǭ}_{22} & 0\\ 0 & 0 & {Ǭ}_{66} \end{array}\right)\left(\begin{array}{c} {ɛ}_{1}\\ {ɛ}_{2}\\ \gamma_{12} \end{array}\right)$$

where

(4) $${Ǭ}_{11}=\frac{E_{1}}{1-\nu_{12}\nu_{21}},\;{Ǭ}_{22}=\frac{E_{2}}{1-\nu_{12}\nu_{21}},\;{Ǭ}_{12}=\frac{\nu_{12}E_{2}}{1-\nu_{12}\nu_{21}}\;\mathrm{and}\;{Ǭ}_{66}=G_{12}$$

In Equation (4), *E*_1_, *E*_2_, *ν*_12_, *ν*_21_, and *G*_12_ are the material parameters of Young’s modulus, Poisson’s ratio, and shear modulus in the ply direction, such as a unidirectional ply.

The composite material is considered anisotropic. Since the stiffness matrix is symmetric, the laminate elastic behavior can be written with 18 material stiffness moduli (6 terms per 3 × 3 matrix, *A*, *B*, and *D*). These matrix moduli are not independent because they are active functions of the ply directions [*θ_k−_*_1_,..., *N*]. However, the six following linear relationships together reduce the number of moduli to be considered to 12 for any normal composite laminate,

(5) $$\begin{array}{c} A_{66}=\frac{1}{2}(A_{11}+A_{22})+h(\frac{1}{2}{(Ǭ}_{11}+{Ǭ}_{12})+{Ǭ}_{16}),\\ A_{12}=A_{66}+h({Ǭ}_{12}+{Ǭ}_{66}),\;B_{66}=\frac{1}{2}(B_{11}+B_{22}),\;B_{12}=B_{66}\\ D_{66}=\frac{1}{2}(D_{11}+D_{22})+\frac{h^{3}}{12}(\frac{1}{2}{(Ǭ}_{11}+{Ǭ}_{22})+{Ǭ}_{16})\\ D_{12}=D_{66}+\frac{h^{3}}{12}{(Ǭ}_{12}-{Ǭ}_{66}) \end{array}$$

**Figure 4:**
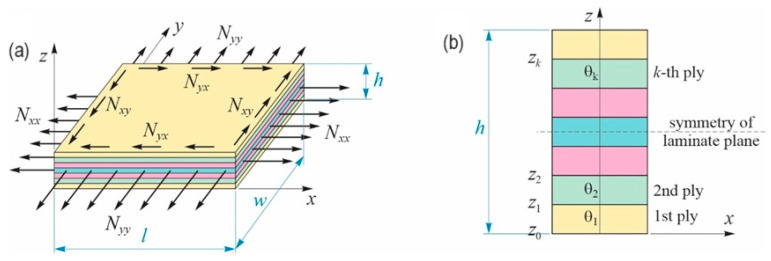
(**a**) Composite laminate general loading configuration. (**b**) Laminate stacking sequence layout.

Figure 4a shows a general loading case of a composite plate with *l*, *w* and *h* in length, width, and thickness, respectively, while Figure 4b presents the laminate stacking sequence layout. 

The laminate will be modeled as orthotropic (*D*_16_ = *D*_26_ = 0), assuming that it can buckle into *m* and *n* halfwaves in the *x-* and *y*-directions when the load amplitude factor reaches a value *λ_wb_* given by the following equation [26],

(6) $$\frac{\lambda_{wb}}{\pi^{2}}=\frac{D_{11}{\left(m/l\right)}^{4}+2(D_{12\;}+2D_{66\;}){\left(m/l\right)}^{2}{\left(n/w\right)}^{2}+D_{22}{\left(n/w\right)}^{4}}{N_{x}{\left(m/l\right)}^{2}+N_{y}{\left(n/w\right)}^{2}+N_{xy}(mn/lw)}$$

where *λ_wb_* is the critical buckling amplitude factor, which depends on the (*m*, *n*), laminate dimensions and the loading case. The buckling at a certain margin *M_b_* can be described by the following relationship,

(7) $$M_{b\;}=(\lambda_{wcb}-1)\times 100\%,\;with\;\lambda_{wcb}=\underset{m,n}{\min}\left(\lambda_{wb}\right)$$

By employing a multi-criterion approach like the Hasin method [26], our method includes the updated failure criteria that allow for the differentiation of fiber failure (*FF*) and laminate matrix failure (*MF*) in both tension and compression modes for each individual layer,

(8) $$Fiberfailure\left(FF\right):\left\{\begin{array}{c} f_{1}^{+}={\left(\frac{\delta_{11}}{x_{t}}\right)}^{2}\;=1,\;if\;\delta_{11}\geq \;0\;\\ f_{1}^{-}={\left(\frac{\delta_{11}}{x_{c}}\right)}^{2}=1,\;if\;\delta_{11}<0\; \end{array}\right.$$

(9) $$Matrix\;failure\;\left(MF\right):\left\{\begin{array}{c} f_{2}^{+}={\left(\frac{\delta_{22}}{Y_{t}}\right)}^{2}+{\left(\frac{\tau_{12}}{S_{c}(1-p\delta_{22}}\right)}^{2}\;=1,\;if\;\delta_{22}\geq \;0\;\\ f_{2}^{-}={\left(\frac{\delta_{22}}{Y_{c}}\right)}^{2}+{\left(\frac{\tau_{12}}{S_{c}(1-p\delta_{22}}\right)}^{2}\;=1,\;if\;\delta_{22}<0\; \end{array}\right.$$

The *p* coefficient enables an accurate description of the reinforcement observed in experimental scenarios, specifically concerning transverse compression and in-plane shear. Here, *X_t_*, *X_c_*, *Y_t_*, *Y_c_*, and *Sc* represent the longitudinal tension and compression strengths, the transverse tension and compression strengths, and the in-plane shear strength, respectively.

(10) $$M_{f}=\left(1/\underset{k,\mathrm{mode}}{\min}(f_{mode}^{\left(k\right)})-1\right)\times 100\%$$

**Figure 5:**
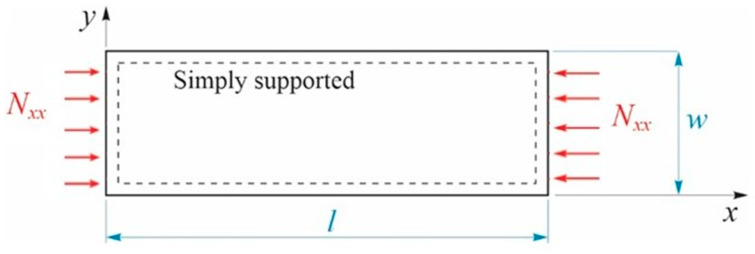
Loading and boundary conditions.

In our case, the plate will be simply supported on all edges and the applied membrane load unidirectional, with *N_xx_* = 250 *N*, as shown in Figure 5.

### 2.2. Design Recommendations 

In this study, we address the optimization of the laminate stacking sequence (LSS) using a multi-objective method. Several important guidelines were considered for the preliminary design of the LSS based on previous laboratory tests and analyses. Previous research [27] has highlighted the importance of balancing the stacking sequence, such as having an equal number of +*θ* and −*θ*-plies and ensuring the symmetry of the laminate about the midplane [28,29], which helps to avoid shear–extension coupling (*A*_16_ = *A*_26_ = *0*) and extension–bending coupling (*B_ij_* = *0*). To minimize the propagation of the laminate matrix (contiguity constraint), it is necessary to avoid using plies with the same orientation and thickness. Additionally, it is important to ensure that two consecutive ply directions do not differ by more than 45° to prevent edge delamination (disorientation constraint). For design aspects where material strength is critical, it is advisable to use a homogeneous LSS. However, when employing plies with angles such as +*θ* and −*θ*-plies, it is recommended to keep them close together. This type of design helps in reducing the effect of bending–twisting coupling, specifically *D*_16_ and *D*_26_. The detailed LSS can be found in [30]. Based on this fundamental overview of the discussed LSS design rules, the elastic characteristics of a symmetric and balanced laminate can be characterized by six stiffness modules, *A*_11_, *A*_22_, *D*_22_, *D*_66_, *D*_26_, and *D*_16_, which need to be minimized.

## 3. Optimization of Design Statement

To optimize the design of the LSS, various specimen boundary conditions need to be considered, including compression and tensile load. The optimization problem can be defined in the following manner:

- Design variables: the ply orientation such as (*θ_k=_*_1_,…, *N*).
- Objectives: minimizing the total number of plies (*N*); minimizing the total weight of the laminate; minimizing the inverse reserve factor; and minimizing the total deformation load multiplier.
- Constraints: the inverse reserve factor is (IRF < 1) and total deformation load multiplier (DLM > 1).
- Fixed parameters: the material, specimen dimensions, boundary conditions, and ply angle discretization is 0°, 30°, 45°, 60°, and 90°.

The inverse reserve factor is a measure of the safety margin in a design, calculated as the ratio of the applied load to the ultimate load capacity. It represents how much the applied load exceeds the strength of the structure. If the inverse reserve factor value is greater than 1, the laminate fails [31]. With the total DLM, designers can estimate the overall deformation or strain in the composite laminate and ensure that it remains within acceptable limits to avoid structural failure or performance issues.

### 3.1. Optimization Methodology

Multi-objective genetic algorithms (MOGAs) are optimization techniques inspired by the principles of Darwinian evolution. These methods are used to solve optimization problems with multiple seemingly conflicting objectives. MOGAs mimic natural selection, crossover, and mutation processes to generate a set of optimum solutions known as the Pareto front, which represents the trade-offs between different objectives [32]. ANSYS Workbench offers tools and interfaces to integrate MOGAs into the optimization process. These tools enable users to define objective functions, constraints, and design variables. MOGAs can then be employed to explore the design space and identify the Pareto front, which represents the set of optimum trade-off solutions [33]. The advantages of the selected optimization methodology (MOGAs in ANSYS Workbench) are that MOGAs can quickly explore a wide range of design possibilities and identify the optimum trade-off solutions. MOGAs enable engineers to consider multiple objectives simultaneously and find a set of solutions that represent the trade-offs between these objectives. The Pareto front generated by MOGAs provides engineers with a comprehensive understanding of the design trade-offs, enabling them to make informed decisions.

### 3.2. Geometrical Model and Analysis

Geometrical models are mathematical representations of geometric shapes and structures used to study and analyze various aspects of geometry. The composite laminate dimensions employed were length *l* = 140 mm, width *w* = 12 mm and thickness *h* = 0.15 mm, as per the ASTM standards. The mechanical properties are given in Table 1.

As already mentioned, the plate was simply supported on all edges and the applied load was unidirectional, with *N_xx_* = 250 *N*. For the analysis, the shell elements were chosen, and the number of nodes and elements was calculated based on the sweep mesh. Since the bidirectional fabric was very thin, 0.15 mm, the total laminate was 2 mm thick. The contiguity constraint in this context refers to a minimum of two adjacent layers having the same orientation. The problem was defined as minimizing the total number of plies, minimizing the total weight of the laminate, minimizing the inverse reserve factor (IRF < 1), and minimizing the total deformation load multiplier. The LSS used as the reference design comprised 16 plies, [45/−45/90_2_/0_3_/90_2_/0_3_/90_2_/−45/45].

### 3.3. Manufacturing of Composite Laminate

**Figure 6:**
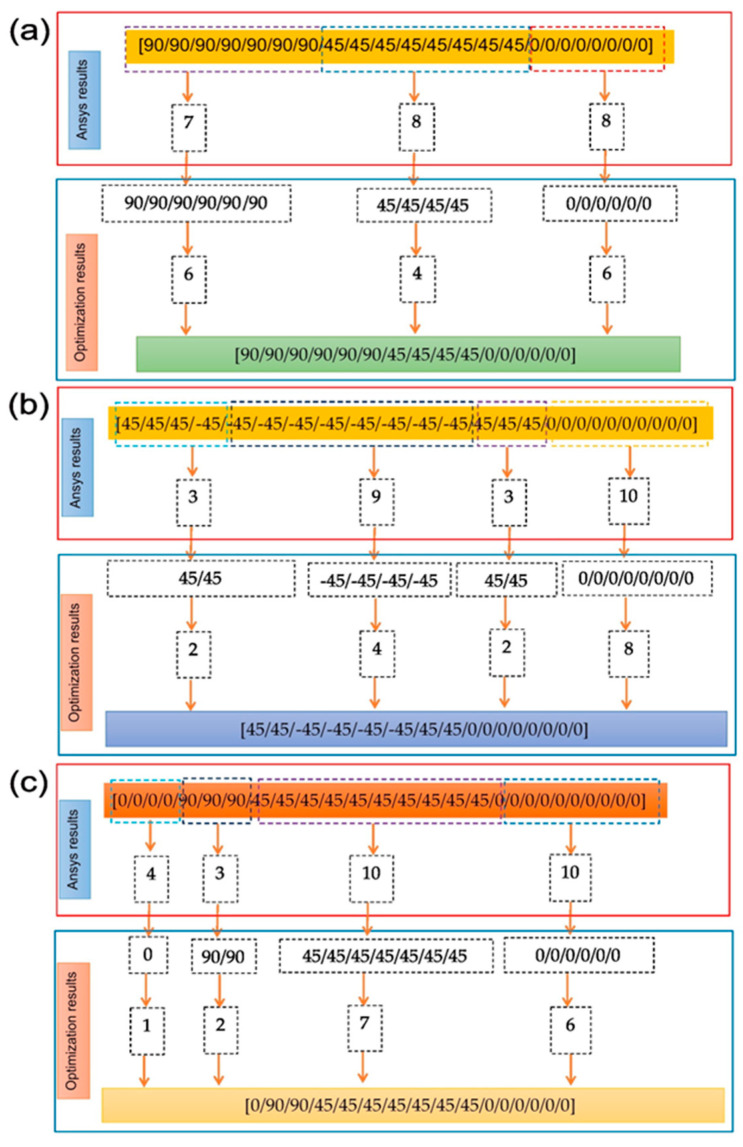
Optimization strategy (**a**) Laminate 1, (**b**) Laminate 2, (**c**) Laminate 3.

In the current study, based on the optimum LSS results, a laminate was fabricated with various configurations using commercially available bidirectional woven E-glass fiber with a thread count of 16 × 15 (i.e., 16 and 15 yarns in the warp and weft directions, respectively, per centimeter, -GF-22-100-100) and LB2 epoxy bio-resin (EP-LB_10) as reinforcement. The E-glass fiber had an area density of 100 g in a 2 × 2 twill woven pattern and a material thickness of 0.15 mm. The resin and hardener were mixed in a ratio of 100:27. The optimization strategy is shown in Figure 6.

E-glass epoxy laminates were manufactured using the hand-layup technique with a vacuum bagging process, incorporating various ply orientations in the LSS. The LSS design is categorized as follows: the Ansys analysis yielded stacking sequence results comprising 24 plies with the sequence [90_7_/45_8_/0_8_], 25 plies with the sequence [45_3_/−45_9_/45_3_/0_10_], and 27 plies with the sequence [0_4_/90_3_/45_10_/0_10_]. After the Ansys results, we systematically reduced the number of plies to attain the specified thickness for mechanical testing, adhering to ASTM testing recommendations. The design methodology employed for Laminate 1 involved transitioning from [90]_7_ to [90]_6_, [45]_8_ to [45]_4_, and [0]_8_ to [0]_6_. This identical methodology was consistently applied to Laminate 2 and Laminate 3. Consequently, the final optimized stacking sequences are as follows: Laminate 1 ([90_6_/45_4_/0_6_]), Laminate 2 ([45_2_/−45_4_/45_2_/0_8_]), Laminate 3 ([0/90_2_/45_7_/0_6_]), and the reference laminate (Laminate 4) ([45/−45/90_2_/03/90_2_/03/90_2_/−45/45]). The optimized LSS results were correlated with the reference stacking sequences, which were used for the initial optimization of the research work. 

**Figure 7:**
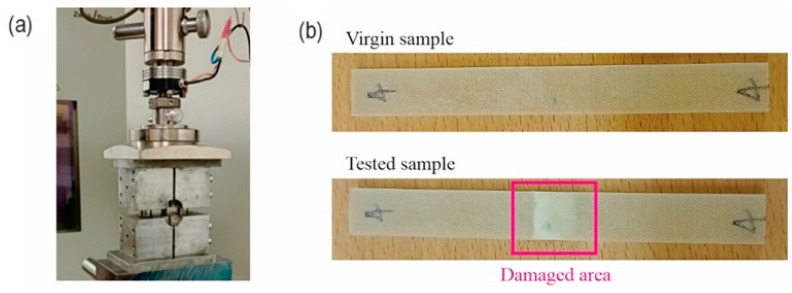
(**a**) Experimental setup, (**b**) virgin and damaged samples.

After successfully fabricating the laminates from 1 to 4, they were left to cure for 48 h. Once cured, the laminates were trimmed to the desired dimensions according to ASTM D 3410, using a Proxxon- PRN27070, 7000 rpm, 220/240V AC, working table 300 × 300 mm table saw. The specimens’ dimensions were a length of 110 mm, a width ranging from 12.10 mm to 12.30 mm, and a thickness range from 2.85 mm to 4.30 mm. The universal testing machine (Zwick/Z050, Zwick Roell, Ljubljana, Slovenia, guided by Test Xpert V.12.0) with a crosshead speed of 2 mm/min. This machine has the capability to perform traction, compression, and bending tests effortlessly, allowing for easy assembly and disassembly of jaws. Additionally, it features a load cell with a capacity of 5 KN and offers a range of loading speeds from 1 to 400 mm/min, which are automatically regulated. The mechanical characterization setup is shown in Figure 7a. In total, 20 samples were fabricated and tested following ASTM standard (ASTM D3410). A virgin sample from Laminate 1 and the result of mechanical loading which leads to damage of the middle area are shown in Figure 7b.

### 3.4. Fracture Analysis of Bidirectional Laminate 2 and 3 ([0–90/±45/0–90], [+45/−45/0])

**Figure 8:**
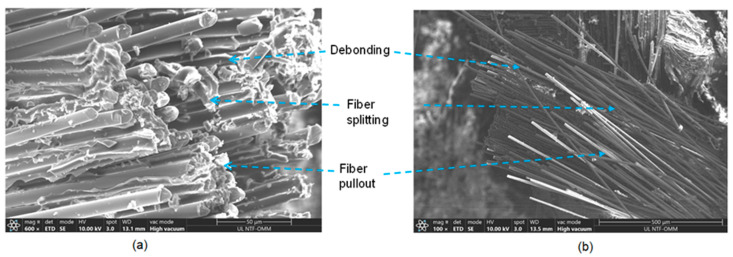
SEM images of E-glass fibers (**a**) fracture surface of E-glass fiber and (**b**) fracture surface of E-glass fibres.

In order to assess the adhesion between the fiber matrix and the composites, E-glass fibers were investigated using a Thermo Scientific scanning electron microscope (SEM, Thermo Fisher scientific, Ljubljana, Slovenia), Quattro S with ULTIM MAX. The SEM samples were coated with carbon using a sputter coating technique. The technical specifications for carbon evaporation included the use of high-purity carbon fiber thread, grade CT4. The carbon thread had a diameter of 0.8 mm and weighed 0.4 g/m. Additionally, Laminate 2 and 3 specimens were analyzed, and the fracture surfaces of the composites resulting from compression tests were also examined using SEM. Figure 8 presents the fracture surfaces of the E-glass fiber composite Laminates 2 and 3. The fiber was debonded from the matrix, and the fibers were split and pulled toward ±45°. The SEM results are presented in Figure 8a,b.

**Figure 9:**
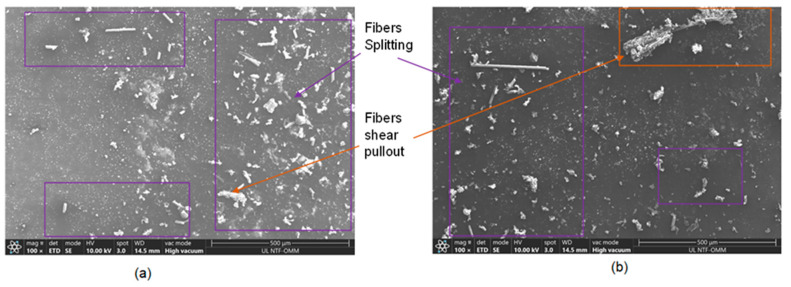
Fiber and matrix failure under compression load (**a**) fibers splitting and (**b**) fibres shearing pullout.

Figure 9 shows the standard fracture surface, illustrating the compression loads on Laminates 2 and 3. Their failure was distinguished by the shearing of the matrix fibers and the splitting of the fibers. Additionally, there were instances of fiber pullout in the ±45° direction. The presence of significant matrix cracks, resulting from the shearing effect, is a distinct criterion for failure in E-glass bio-epoxy composite laminates under compression load. These cracks are highlighted by the rectangles in Figure 9a,b.

**Figure 10:**
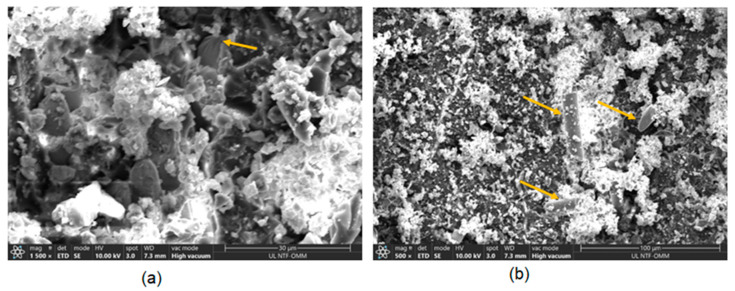
Spherulitic failure under compression load (**a**) fibers spherulitic failure and (**b**) fibres failure.

The spherulitic failure illustration is shown in Figure 10. The spherulitic MF in Figure 10a indicates the occurrence of compressive force around the fiber, as highlighted by the arrow. This compressive zone is more prone to brittleness compared to the surrounding matrix. Figure 10b demonstrates the tendency for matrix cracking and *FF* under compressive load, as indicated by the arrow.

## 4. Results and Discussion 

The MOGAs method is a variant of the popular NSGA-II (Non-dominated Sorted Genetic Algorithm-II) based on controlled elitism concepts. It supports multiple objectives and constraints and aims at finding the global optimum, generating 200 samples initially, 50 samples per iteration, and finding three candidates. These results can be further processed to present the designer with a sorted collection of solutions. Optimum solutions are sorted in terms of minimum buckling margins, such as inverse reserve factor, maximizing the total weight of the laminate, and minimizing the buckling load factor. The results displayed in Table 2 and Table 3 were chosen from the set of optimum solutions generated by the optimization algorithm after a single run comprising 200 evaluations for different combinations of plies and ply angle constraints. The ply-stacking sequences considered were 0°, ±30°, ±45°, ±60°, and 90°. The highest inverse reserve factor led to the failure of the laminate. In Table 3, the results address the homogeneity constraints, and these types of solutions can avoid bending and twisting.

The results presented in Table 2 and Table 3 were achieved by enforcing the balance and symmetric constraints. For Table 2 and Table 3, the optimization process involved utilizing ply orientations {0°, ±45°, 90°}, {0, ±45°, 0°}, {90°, ±45°, 0°}, {±45°, 0°, 90°} and {0°, ±30°, ±45°, ±60°, 90°} as a set of choices. However, in Table 2, the different ply orientations were extended to {0°, ±30° ± 60°, and 90°} and a number of plies were noted, such as 24, 25, 27, 30, 34, 45, 45, 57, and 47. To minimize the LSS, which is one of the current constraints of the design guidelines, the results obtained in Table 2 were the lowest among all the obtained results. The maximum inverse reserve factor value is 0.090; however, the number of plies decreased compared to Table 3, specifically down to 24 plies. Simultaneously, the total weight of the laminate also slightly increased. The buckling load factor also has an impact on the laminate structure. In Table 3, a lower load factor of 2.3960 was observed. The total weight of the laminate Table 3 was recorded, with a value of 6.17 × 10^−6^ kg/m^3^ at 36 and 45 plies. Careful attention was paid to the number of plies, weight, and the IRF value. The number of plies remained the same (25 and 26) in both tables. However, in Table 3, the weight of the laminates slightly increased due to the additional plies (25 to 52). Notably, the load factor also increased from 2.515 to 17.755.

The contrast between Table 2 and Table 3, along with various other tables, underscores the potential advantages that arise from incorporating new ply orientations in comparison to the traditional {0°, ±45°, 90°} arrangement. This phenomenon becomes more apparent with the inclusion of disorientation and homogeneity constraints, as these rules impose greater restrictions on {0°, ±45°, 90°} laminates compared to {0°, ±30°, ±60°, 90°} laminates. Additionally, the mechanical properties of four different composite laminates with different ply orientations (Laminate 1, Laminate 2, Laminate 3, and a reference Laminate 4) were investigated. The obtained mechanical compression test results are shown in Table 4. The properties analyzed include the maximum force (F_max_), displacement at F_max_ (dL), thickness, width, cross-sectional area (Area), and ultimate compressive strength (σc). Laminate 1 exhibited an F_max_ of 8370.988 N with a corresponding displacement of 1.528346 mm. The laminate had a thickness of 4.2 mm, a width of 12.16 mm, and an area of 51.072 mm^2^. The ultimate compressive strength was 163.9056 MPa. Similarly, Laminate 2 displayed an F_max_ of 5777.181 N and a displacement of 1.457818 mm. It had a smaller thickness of 3.2 mm, a width of 12.1 mm, and an area of 38.72 mm^2^. The ultimate compressive strength for Laminate 2 was 149.204 MPa. Laminate 3 demonstrated a F_max_ of 5167.207 N and a displacement of 1.311693 mm. It had a thickness of 3.55 mm, a width of 12.25 mm, and an area of 43.4875 mm^2^. The ultimate compressive strength was determined to be 118.8205 MPa. The reference Laminate 4 had an _Fmax_ of 7868.818 N and a displacement of 1.75447 mm. 

**Figure 11:**
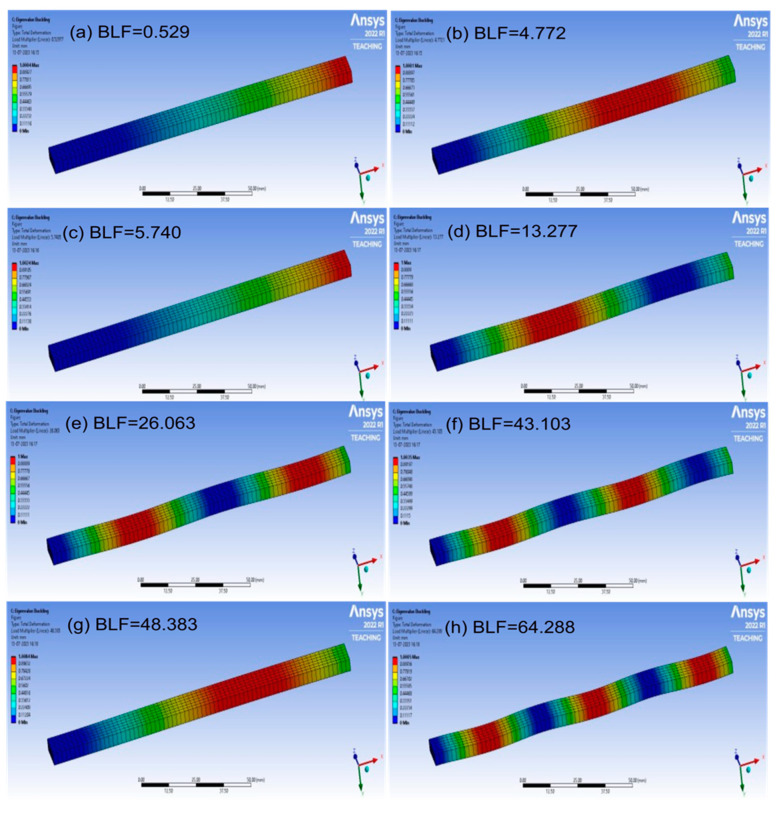
The buckling analysis results, mode shapes from (**a**–**h**) for Laminate 1.

**Figure 12:**
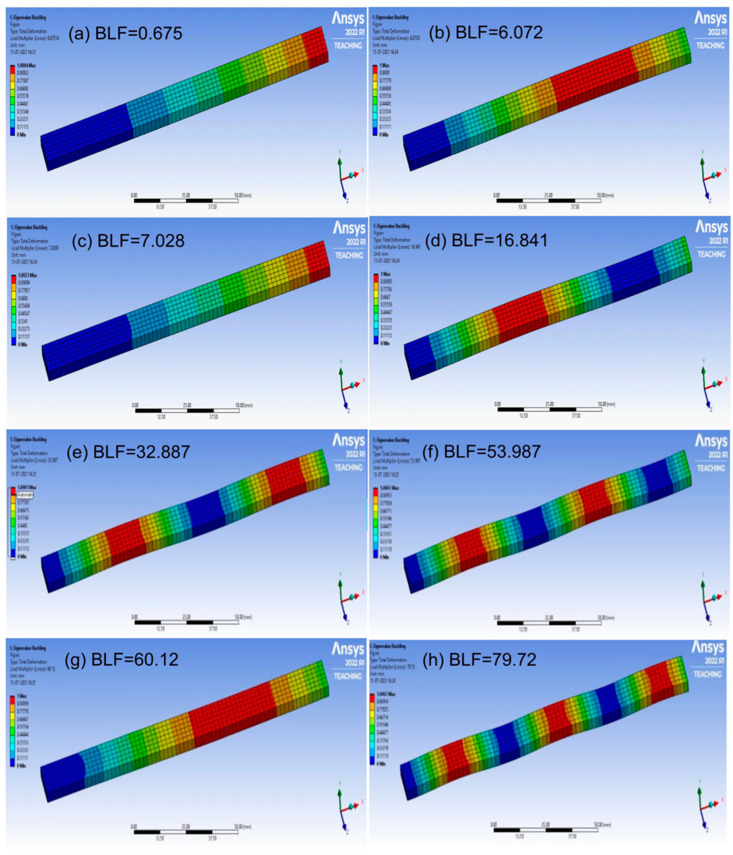
The buckling analysis results, mode shapes from (**a**–**h**) for Laminate 2.

**Figure 13:**
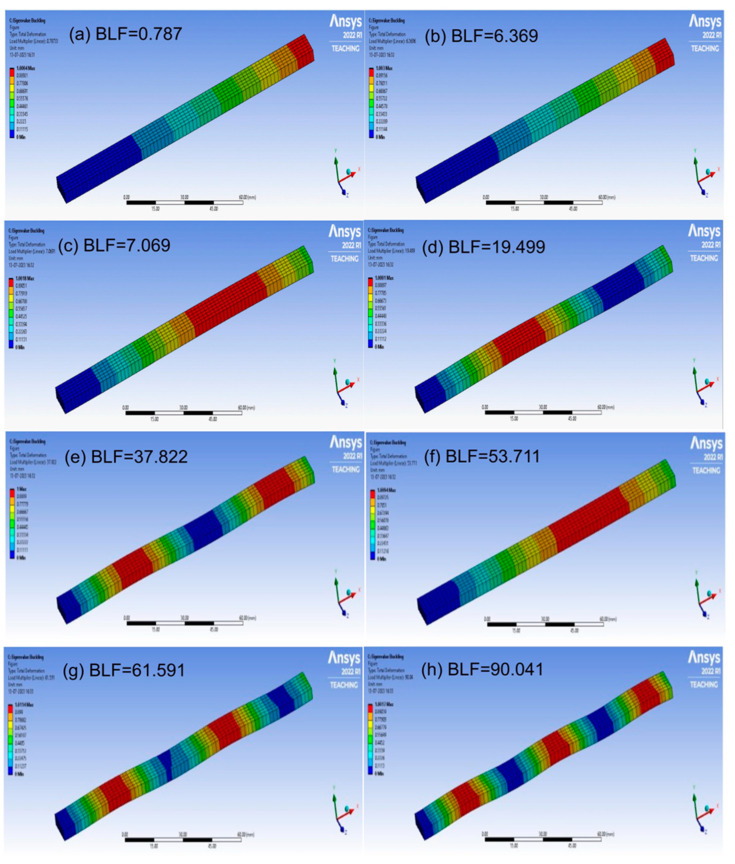
The buckling analysis results, mode shapes from (**a**–**h**) for Laminate 3.

**Figure 14:**
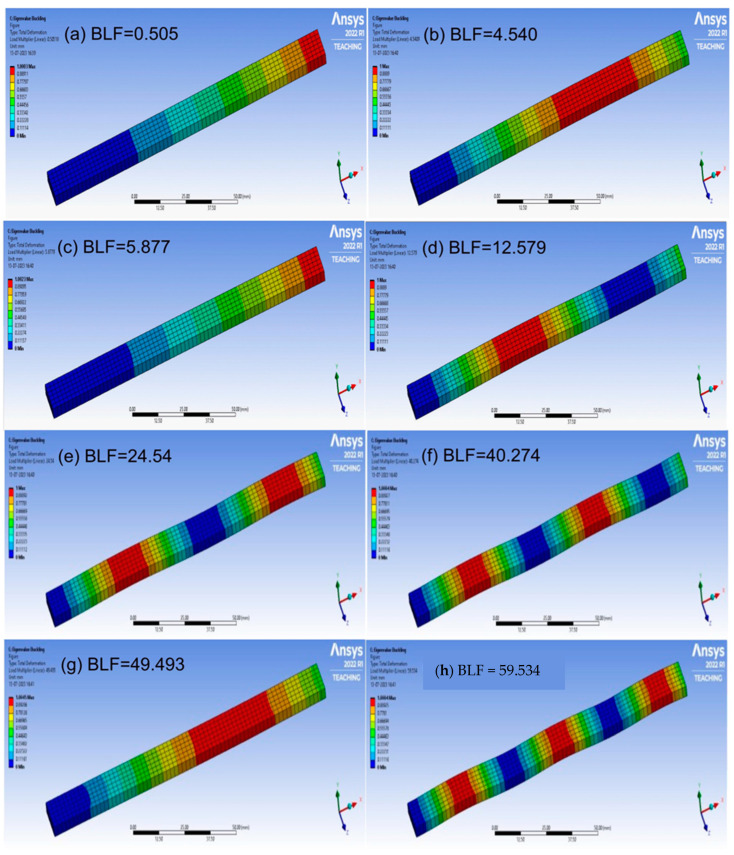
The buckling analysis results, mode shapes from (**a**–**h**) for Laminate 4.

The buckling of the composite plates is a very complicated subject, and more details can be seen in references [34,35,36,37,38,39]. The buckling analysis was successfully carried out; the analysis results are shown in Figure 11, Figure 12, Figure 13 and Figure 14, respectively. The presented data represent the buckling mode shapes of a composite laminate with various stacking sequences. The analysis includes mode shapes at different angles, each characterized by a specific amplitude. Understanding these mode shapes is crucial for assessing the laminate’s behavior under various loading conditions, particularly in relation to buckling phenomena. The buckling mode shapes and corresponding amplitudes provide valuable insights into the critical buckling behavior of the composite laminate under different orientations. 

**Figure 15:**
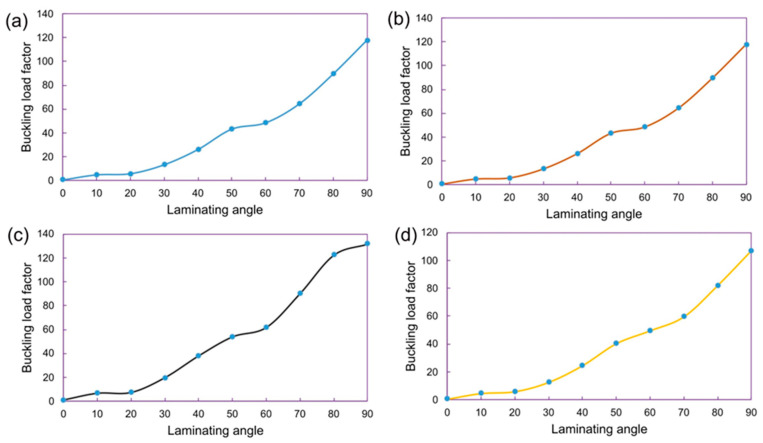
Buckling analysis results, buckling load factor vs. laminating angle plots at various stacking sequences, (**a**) Laminate 1, (**b**) Laminate 2, (**c**) Laminate 3 and (**d**) Laminate 4 (reference laminate).

The buckling load factor vs. laminating angle data are provided in Figure 15, which shows valuable insights into the buckling behavior of the composite laminate. Understanding how the buckling load factor varies with the laminating angle is crucial for designing laminates with improved stability and resistance to buckling failure.

**Figure 16:**
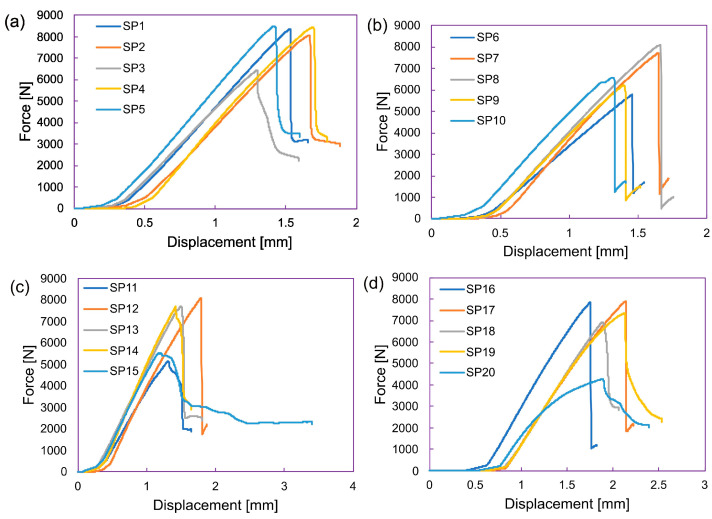
Compression test results, force vs. displacement plots at various stacking sequence, (**a**) Laminate 1, (**b**) Laminate 2, (**c**) Laminate 3 and (**d**) Laminate 4 (reference laminate).

It possessed a thickness of 4.15 mm, a width of 12.21 mm, and an area of 50.6715 mm^2^. The force and displacement plots of Laminates 1 to 4 are presented in Figure 16.

The information gathered is displayed in Table 5, and composite laminates are analyzed using Ansys and Classic Laminate Theory (CLT), followed by ply orientation optimization to improve structural performance. Inverse reserve factor (IRF), ply-stacking sequence, buckling load factor (BLF), laminate weight, and critical buckling load (N_cr_) are among the criteria that were considered. According to the findings of both theories, the Ansys data show variations in the IRF, critical buckling load (N_cr_), and BLF in comparison to CLT for a variety of stacking sequences, including [(90)_8_/(45)_8_/(0)_8_] and [(45)_3_/(−45)_9_/(45)_3_/(0)_10_]. Differences between Ansys and CLT results emphasize how crucial it is to use more advanced numerical techniques to accurately represent the complexities of composite behavior in the real world. Furthermore, the safety margin against failure is indicated by the IRF values derived from Ansys simulations. As seen by Laminates 1 and 3, lower IRF values imply a closer proximity to failure. The stability of the laminate under applied loads is shown by the BLF. In comparison to other configurations, Laminate 2 shows greater BLF-Ansys values, indicating improved resistance to buckling. This highlights how important it is to have optimal ply orientations to improve the structural integrity of composite laminates. A crucial measure that indicates the load at which buckling becomes critical is the critical buckling load (N_cr_), which is another important component. Laminate 3 exhibits a higher N_cr_-Ansys, indicating superior resistance to buckling under the stated conditions. Its ply-stacking sequence is [(0)_1_/(90)_2_/(45)_7_/(0)_6_]. To strike a compromise between structural performance and weight, the optimization efforts in ply orientation ultimately determine the weight of the laminate. Different ply-stacking sequences are shown in the optimal composite laminates (Laminate 1 through Laminate 4), which causes changes in laminate weight. For example, Laminate 3 strikes a good compromise between structural performance and weight. The shortcomings of oversimplified theories in precisely forecasting the behavior of composite laminates are demonstrated by the comparison of Ansys and CLT results. Ply orientations that have been tuned demonstrate how customized designs can improve structural performance.

## 5. Reliability of Current Work

**Figure 17:**
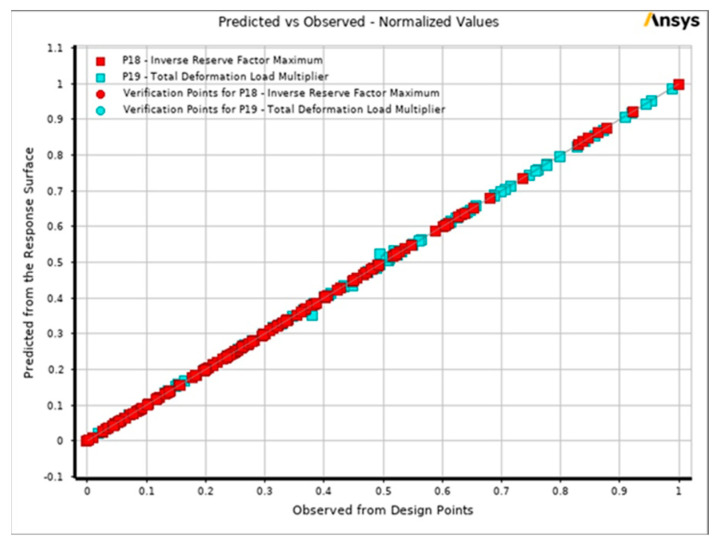
Response surface optimization predicted vs. observed values.

In this discussion, the algorithm’s effectiveness is examined by considering the average number of evaluations needed to achieve a specific level of reliability for discovering a laminate that demonstrates optimum performance [40,41,42]. The reliability is determined by conducting 200 optimization runs consisting of 200 samples initially, 50 samples per iteration, and finding three candidates. A solution was deemed optimum when it effectively minimized the buckling factor. Figure 17 illustrates the comparison between the predicted and observed values of the maximum inverse reserve factor and buckling load factor across different design points.

The summarized results obtained from the response surface optimization values are presented in Table 6.

As anticipated, considering the computational cost associated with the constraints, the highest achievable reliability was attained after 200 evaluations. The experiment followed a central composite design, which was automatically chosen. This approach yielded the optimum results for the optimization.

**Figure 18:**
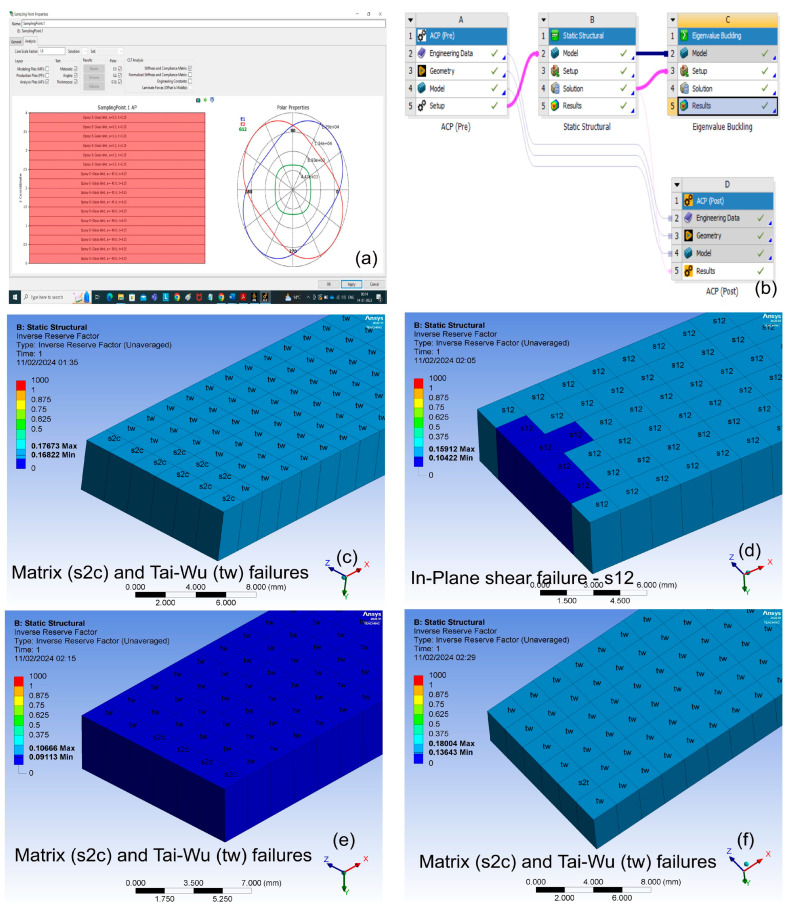
Composite laminates configuration and Identified failure criteria, (**a**) Plies construction, (**b**) Ansys setup, (**c**) Laminate 1, (**d**) Laminate 2, (**e**) Laminate 3 and (**f**) Laminate 4 (reference laminate).

Figure 18 shows that Matrix failure is a recurring subject in Laminate 1, Laminate 3, and Laminate 4, indicating potential material or production concerns. Tai-Wu failure is detected in Laminates 1, 3, and 4, indicating a persistent issue with stress distribution or design issues. Laminate 2 exhibits in-plane shear failure, which may imply unique bonding or load distribution issues specific to this laminate arrangement.

## 6. Conclusions and Future Work

A novel optimization approach was devised specifically for laminates with composite stacking sequences. This strategy effectively addresses two key industrial demands. Firstly, it enables the handling of many load factor cases, thereby increasing the number of objective functions and constraints involved. Secondly, it addresses the diverse and intricate concepts and rules governing stacking sequences. Utilizing the MOGAs approach as a foundation, the evolutionary algorithm created in this study demonstrated a high level of efficiency in managing numerous objective functions and constraints, reaching several hundred in number. In regard to the conception rules, they were integrated within the evolution and reproduction operators of the genetic algorithm. Consequently, only permissible solutions that adhered to these rules were considered during the process. This proposed strategy is well suited for post-processing tasks, facilitating sorting and further design reduction efforts.

The post-processing phase encompassed three criteria: minimizing the inverse reserve factor, minimizing the load factor, and ultimately minimizing the number of plies. When compared to conventional design methods, the proposed strategy yielded notable enhancements across these abovementioned criteria. The findings underscored the potential advantages associated with introducing new ply orientations as opposed to the classical {0°, ±45°, 90°} arrangement. Within this study, the optimum results are presented in Table 2, where the minimum number of plies is 24, the IRF value is 0.1044, the weight of the laminate is 5.73 × 10^−6^ kg/m^3^, and the buckling load factor is 2.3960. The ultimate compressive strength for the reference laminate was 155.2908 MPa. Comparing the results, Laminate 1 exhibited the highest F_max_ and ultimate compressive strength, while Laminate 3 had the lowest values in both parameters. Laminate 2 had the smallest thickness and area, resulting in lower F_max_ and ultimate compressive strength compared to the other laminates. Surface fracture analysis was also carried out and identified fiber and matrix cracks. Engineers and designers can utilize this information to optimize the stacking sequence and orientation of plies to enhance the laminate’s resistance to buckling and improve overall structural performance in real-world applications.

In summary, the analysis emphasizes the importance of advanced numerical simulations and optimization techniques in tailoring composite laminates for specific performance criteria, paving the way for improved structural efficiency in various engineering applications. These findings suggest that the material composition and design of the laminates significantly influence their mechanical properties. These outcomes can be deemed the optimum stacking sequence, making them valuable for future applications in UAV and automobile structures.

## Figures and Tables

**Table 1:** Mechanical properties of bidirectional fiber.

*E*_1_ (GPa)	*E*_2_ (GPa)	*ν*_12_	*G*_12_ (GPa)	
45	10	0.3	5	
***X*_t_** **(MPa)**	***X*_c_** **(MPa)**	***Y*_t_** **(MPa)**	***Y*_c_** **(MPa)**	***S*_c_** **(MPa)**
780	480	31	10	60

**Table 2:** Optimum ply-stacking sequence results at various configurations (0°/±45°/90°).

	Plies Quantity	Ply-Stacking Sequence	Inverse Reserve Factor (IRF)	Buckling Factor/Load Multiplier	Weight of the Laminate
stacking sequenc {0°,±45°,90°}	24	[90_7_/45_8_/0_8_]	0.1044	2.3960	5.73 × 10^−6^
	25	[45_3_/−45_9_/45_3_/0_10_]	0.1115	2.5453	5.73 × 10^−6^
	27	[0_4_/90_3_/45_10_/0_10_]	0.04075	4.6152	5.75 × 10^−6^
Stacking sequence {0°,±45°,0°}	30	[−45_8_/90/45/−45_6_/45_11_/0_3_]	0.0901	5.5743	5.88 × 10^−6^
	34	[0_2_/−45_5_/90_2_/−45_8_/45_14_/0_3_]	0.0582	6.4597	5.91 × 10^−6^
	45	[0_6_/45_9_/−45_9_/45_15_/0_6_]	0.0305	15.5221	5.83 × 10^−6^
stacking sequence {±45°,0°,90°}	45	[45/90_2_/0_11_/45_8_ /0_4_/90_19_]	0.0818	10.4888	6.64 × 10^−6^
	57	[−45_9_/45_32_/0_6_/90_10_]	0.0719	14.7583	6.61 × 10^−6^
	47	[90_3_/45_18_/−45_10_/0_6_/90_10_]	0.1236	10.0268	6.73 × 10^−6^

**Table 3:** Optimum ply-stacking sequence results at various configurations (90°/±45°/0° and (0°/±30°/±60°/90°).

	Plies Quantity	Ply-Stacking Sequence with Symmetric and Unbalanced Constraints and Homogeneity Constraints	Inverse Reserve Factor (IRF)	Buckling Factor/Load Multiplier	Weight of the Laminate
Stacking sequence {90°,±45°,0°}	52	[0_11_/−45_11_/0_3_/−45_16_/45_11_]	0.0479	17.7556	6.12 × 10^−6^
	45	[−45_5_/0_6_/−45/0_4_/90_10_/−45_14_/45_5_]	0.0698	12.1819	6.17 × 10^−6^
	36	[90_7_/45_7_/90_2_/0_3_/−45_12_/45_5_]	0.0783	7.9316	6.17 × 10^−6^
Stacking sequence {0°,±30°±60°,90°}	26	[−60_8_/−30_5_/30_7_/0_6_]	0.1009	2.5155	5.73 × 10^−6^
	25	[−20_2_/60_9_/30_7_/0_7_]	0.0889	2.6258	5.70 × 10^−6^
	27	[30_4_/90_4_/−30_7_/30_12_]	0.0639	3.579	5.75 × 10^−6^
Stacking sequence {0°,±30°±60°}	26	[0_6_/30_14_/0_6_] s	0.0333	4.6113	5.67 × 10^−6^
	25	[30_2_/60_9_/30_7_/0_7_]	0.0889	2.6258	5.73 × 10^−6^
	37	[60_2_/0_5_/60_7_/0_7_/−30_7_/30_7_/0_2_]	0.0607	7.1319	5.78 × 10^−6^

**Table 4:** Compression test result.

		F_max_	dL (F_max_)	Tickness	Width	Area	σc
		N	mm	mm	mm	mm^2^	MPa
Laminate 1	SP 1	8370.988	1.528346	4.2	12.16	51.072	163.9056
	SP 2	8074.094	1.665818	4.1	12.1	49.61	162.7513
	SP 3	6448.679	1.296282	4.15	12.12	50.298	128.2094
	SP 4	8430.247	1.685427	4.2	12.1	50.82	165.8844
	SP 5	8500.917	1.414758	4.3	12.12	52.116	163.1153
Laminate 2	SP 6	5777.181	1.457818	3.2	12.1	38.72	149.204
	SP 7	7716.452	1.647232	2.85	12.2	34.77	221.9284
	SP 8	8095.023	1.660723	3.15	12.5	39.375	205.5879
	SP 9	6228.349	1.396224	3.38	12.32	41.6416	149.5704
	SP 10	6576.884	1.312205	3.3	12.27	40.491	162.4283
Laminate 3	SP 11	5167.207	1.311693	3.55	12.25	43.4875	118.8205
	SP 12	8106.793	1.792589	4.35	11.93	51.8955	156.2138
	SP 13	7709.107	1.501875	4.42	11.88	52.5096	146.8133
	SP 14	7708.057	1.421926	4.33	12.28	53.1724	144.9635
	SP 15	5532.686	1.176934	4.33	12.29	53.2157	103.9672
Laminate 4 (Reference laminate)	SP 16	7868.818	1.75447	4.15	12.21	50.6715	155.2908
	SP 17	7907.699	2.141184	4.14	12.12	50.1768	157.5967
	SP 18	6915.887	1.883853	4.31	12.19	52.5389	131.6337
	SP 19	7360.653	2.130509	3.94	12.18	47.9892	153.3814
	SP 20	4275.142	1.881446	3.87	12.08	46.7496	91.44767

**Table 5:** Optimization results at various ply orientations.

		Plies	Ply-Stacking Sequence	Inverse Reserve Factor			Buckling Load Factor			Critical Buckling Load			Laminate Weight
				(IRF-Ansys)	IRF CLT	Error	BLF Ansys	BLF CLT	Error	N_cr_ Ansys	N_cr_ CLT	Error	
Ansys results		24	[(90)_8_/(45)_8_/(0)_8_]	0.104	0.185	0.081	2.396	2.68	0.284	592	672	80	5.73 × 10^−6^
		25	[(45)_3_/(−45)_9_ /(45)_3_/(0)_10_]	0.111	0.300	0.189	2.545	2.73	0.185	636	684	48	5.73 × 10^−6^
		27	[(0)_4_/(90)_3_/(45)_10/_(0)_10_]	0.040	0.084	0.044	4.615	2.73	1.885	1153	684	469	5.75 × 10^−6^
Optimized Plie-orientation results	Laminate 1	16	[(90)_6_/(45)_4_/(0)_6_]	0.176	0.165	0.011	4.772	2.73	2.042	1193	684	509	1.243 × 10^−5^
	Laminate 2	16	[(45)_2_/(−45)_4_/(45)_2_/(0)_8_]	0.159	0.479	0.320	6.072	2.75	3.322	1518	689	829	1.243 × 10^−5^
	Laminate 3	16	[(0)_1_/(90)_2_/(45)_7_/(0)_6_]	0.106	0.107	0.011	6.369	2.85	3.519	1592	712	880	1.243 × 10^−5^
	Laminate 4 (Random orientation)	16	[(45)_1_/(−45)_1_/(90)_2_/(0)_3_/(90)_2_ /(0)_3_/(90)_2_/(45)_1_/(−45)_1_]	0.180	0.086	0.094	4.540	2.69	1.85	1135	673	462	1.243 × 10^−5^

**Table 6:** Response surface optimization results.

	Inverse Reserve Factor	Buckling Load Factor
**Coefficient of Determination (Best Value = 1)**		
Learning Points	1	0.999
Cross-Validation on Learning Points	0.892	0.994
**Root Mean Square Error (Best Value = 0)**		
Learning Points	4.551 × 10^−8^	0.00048274
Verification Points	3.6194 × 10^−7^	0.00039631
Cross-Validation on Learning Points	0.033006	0.0014322
**Relative Maximum Absolute Error (Best Value = 0%)**		
Learning Points	0	12.822
Verification Points	0	2.0451
Cross-Validation on Learning Points	242.51	55.837
**Relative Average Absolute Error (Best Value = 0%)**		
Learning Points	0	1.1931
Verification Points	0	2.0451
Cross-Validation on Learning Points	13.993	4.3063

## Data Availability

Data are contained within the article.
